# Pay Wards in General Hospitals

**Published:** 1895-03-30

**Authors:** 


### THE INSTITUTIONAL WORKSHOP.

### HOSPITAL ADMINISTRATION.

### PAY WARDS IN GENERAL HOSPITALS.

AT the present time the majority of the hospitals are making preparations for holding their annual meetings, if they have not, indeed, already taken place. It is, in fact, the most active period of the year, so far as the hospital governors are concerned, seeing that they are invited to attend the annual meetings, that a copy of the report is sent to them, and that the collector calls for their subscriptions. It follows that if there is one time better calculated than another to arouse and fix the interest of the public in the management of our hospitals, it is the present season. There can be no doubt, having regard to the continuous growth in the population, and the consequent increase in the number of applicants for hospital relief, that it is desirable, if possible, to find new methods of raising income which shall tend to secure a diminution of the abuses which have heretofore marked much of the relief which has been given to the sick poor in this country. In THE HOSPITAL for March 2nd, to which we direct special attention, we gave the history of the rise and progress of the Great Northern Central Hospital and showed that the introduction of the combined free and pay system there had been attended by surprisingly good financial results. The case of the Great Northern Central Hospital proves to demonstration that the people who support hospitals are in favour of pay wards attached to general hospitals. Twenty years ago there was scarcely a hospital in the United Kingdom which admitted patients on payment in any form or shape or way. Now all classes of hospitals take payment from their patients in certain circumstances, and the principle has been generally approved by those who have paid the most attention to its bearings upon medical relief. At the present time, for instance, the general hospitals in London and the provinces derive rather less than 2 per cent. of their income from patients' payments. Those in Scotland derive rather more than 3 per cent., and those in Ireland rather more than 7 per cent. of their income from patients' payments. Of the special hospitals, those in London receive about 6¾ per cent. of their income, those in the provinces 10¾ per cent. of their income, and those in Ireland 6½ per cent. of their income, from same source. Scotch special hospitals, of which there the are very few, receive less than ¼ per cent. from patients' payments at the present time. We mention these facts here, in order to show that the question is not a new one; that the principle it embodies has commended itself to very many hospital authorities, and that whatever its merits or demerits, the pay system has now come within the range of hospital politics, and presses for consideration and settlement.

MEDICAL ASPECTS OF THE QUESTION.

It has been contended by those who have essayed to speak on behalf of the general practitioner of medicine that the admission of pay patients to a general hospital is an abuse of his privileges, and must be withstood to the death. Is this the case? Our answer is No; because, rightly understood and properly applied, the pay system should prove a bulwark for the general practitioner, and should tend more to protect his interests by abolishing the abuse of hospitals than anything which can be mentioned.

The truth is that the general practitioners and others have jumped to the conclusion that the admission of paying patients to a general hospital is the same thing as the taking of payment from a patient in the general wards of a hospital. Now, rightly understood, these two things are wholly apart and distinct from each other. It is impossible for any general hospital in this country to accept payment from a patient in a general ward without doing injustice to the hospital and also to the general practitioner of medicine. This is so, because any hospital which takes payment from patients it admits for treatment must provide in its rules that a certain proportion of those payments shall be devoted towards the cost of medical attendance. It follows further, that the class of patients who are able and willing to pay for their treatment in a hospital, will not, as a rule, consent to be treated in a general ward where there are free beds. On the other hand, when a hospital has been specially designed, like the Great Northern Central, to admit pay patients for treatment within its walls, in such a case the pay system must prove a protection both to the institution and to the general practitioners of medicine, providing always that the rules of admission are properly drawn and enforced. A hospital so designed enables the pay patients to be treated in a separate block or wing, apart from the ordinary wards and work of the hospital. In such circumstances every patient in the pay wing can be properly and rightly treated by his own medical man, upon terms which should prove mutually satisfactory. There can be no question that the regulations at the Great Northern Central Hospital at the present time are for this reason inadequate and inimical to the interests of the hospital, its honorary medical staff, and the great body of medical practitioners in North London. We hope to see the day when these regulations will be more in accord with experience and knowledge.

A general hospital having a separate pay wing to which admission can only be obtained upon conditions which protect the interests of all concerned, would deserve, and would, we believe, receive the liberal support of all thoughtful people. Such a hospital, providing it had the wisdom to take the further step of paying the whole of its medical officers, and abolishing unpaid medical service altogether, would bring about a revolution in hospital administration, and prevent hospital abuse, so far as its own work was concerned for all time. It would do more than this for the combined free and pay system where the whole institution is worked by a medical staff which is adequately paid for its services, involves such an organization as must secure the hearty co-operation of all the general practitioners of the district, which the institution serves, as well as of the residents, who, having the right of admission to the pay wards, and to the attendance, at their option, of their own doctors during their residence therein, would feel constrained to combine with the members of the medical profession and the hospital authorities to prevent anyone from obtaining free medical relief unless entitled to receive it. If a patient be entitled to receive free medical relief, then the volume of that relief should be unlimited and adequate, whilst the relief itself must necessarily be of the best. We commend these considerations to the attention of all who are engaged in the administration of our voluntary hospitals, to the members of the medical profession of all ranks, and to the supporters of hospitals of high and low degree.

### An American Example.

It is the practice in some American hospitals which are economically and wisely administered to present a budget for the coming year, through their treasurer, at the annual meeting, as well as an audited statement of the accounts of the past year. This system enables the treasurer and Finance Committee to report what money will be available during the new year to meet the expenditure. They are thus able to show how many beds out of the number available the governors are justified in declaring free for the ensuing twelve months, and how many it is desirable to allocate as pay beds. By this simple system the finances are readily adjusted and serious deficits are avoided. Of course, in a comparatively new country like the United States, except in a few of the older cities, the invested funds do not represent a very large sum, and the managers of the hospitals have in consequence to raise nearly all their income each year. This circumstance makes for efficiency, because an institution which is mainly dependent upon the free offerings of the charitable has to keep in touch with the public, and to take care that the administration is efficient and economical. In the report of an American hospital just to hand, the regulations as to the admission of paying patients provide that any physician of good standing may, by application to the resident physician, receive permission to treat his own private patients in the pay wards of the hospital. The application for the admission of patients must in every case be accompanied with a written diagnosis of the case. The resident physician then reports the application to the executive committee, and, if approved, arrangements are made for the patient to occupy a private room, on paying the hospital charges, providing he is a private patient of the physician who made the application. The board of trustees reserve the right to suspend the privileges granted to the private practitioner, and for reasons stated, to revoke the privilege of the physician in question to treat patients in the hospital. When there is a pressure upon the beds, in the event of a member of the medical staff and a private practitioner presenting patients at the same time for a private room, the preference is given in all cases to the member of the medical staff. At the monthly meeting of the board of trustees the resident physician presents a return, containing the names of the physicians not members of the staff, who have treated private patients in the hospital, and the number of patients treated by each during the month. These simple rules are found to give every satisfaction to the honorary medical staff, the private practitioners, the patients, and the managers of the hospital.

### The Right and Wrong Systems.

Of course, as we have already said, it is quite impossible that any general hospital in this country could permit a patient in a general ward to be treated there by his own medical man. Such a plan must give rise to all kinds of difficulties, and would break down in practice. On the other hand, where, as at the Great Northern Central Hospital, there is a separate wing to which admission can be obtained without entering the general hospital at all, there should be no difficulty in permitting pay patients to be attended by their private practitioner, at their own option. We feel that the general practitioners of North London have a right to feel aggrieved by their exclusion from the pay wing of the Great Northern Central Hospital, and we hope a fair compromise may be speedily arrived at which will remove every cause of friction, or difficulty of administration, and at the same time do justice to all concerned.

